# Mitochondria Death/Survival Signaling Pathways in Cardiotoxicity Induced by Anthracyclines and Anticancer-Targeted Therapies

**DOI:** 10.1155/2012/951539

**Published:** 2012-03-20

**Authors:** David Montaigne, Christopher Hurt, Remi Neviere

**Affiliations:** Department of Physiology (EA4484), Faculty of Medicine, University of Lille 1, Place de Verdun, 59045 Lille, France

## Abstract

Anthracyclines remain the cornerstone of treatment in many malignancies but these agents have a cumulative dose relationship with cardiotoxicity. Development of cardiomyopathy and congestive heart failure induced by anthracyclines are typically dose-dependent, irreversible, and cumulative. Although past studies of cardiotoxicity have focused on anthracyclines, more recently interest has turned to anticancer drugs that target many proteins kinases, such as tyrosine kinases. An attractive model to explain the mechanism of this cardiotoxicity could be myocyte loss through cell death pathways. Inhibition of mitochondrial transition permeability is a valuable tool to prevent doxorubicin-induced cardiotoxicity. In response to anthracycline treatment, activation of several protein kinases, neuregulin/ErbB2 signaling, and transcriptional factors modify mitochondrial functions that determine cell death or survival through the modulation of mitochondrial membrane permeability. Cellular response to anthracyclines is also modulated by a myriad of transcriptional factors that influence cell fate. Several novel targeted chemotherapeutic agents have been associated with a small but worrying risk of left ventricular dysfunction. Agents such as trastuzumab and tyrosine kinase inhibitors can lead to cardiotoxicity that is fundamentally different from that caused by anthracyclines, whereas biological effects converge to the mitochondria as a critical target.

## 1. Introduction

Cardiotoxicity is a term often used to describe a broad range of adverse effects on heart function induced by therapeutic molecules. These effects may either emerge early in preclinical studies or become apparent latter in the clinical setting after the drug has already been licensed for clinical use. The use of several chemotherapeutics for the treatment of cancer is associated with a risk of cardiovascular complications [1–3]. They present as a defect in cardiac function that can be either symptomatic or not. The classic example of this issue is the use of anthracyclines such as doxorubicin, which is commonly prescribed to treat hematological malignancies and solid tumors [4, 5].

Potential cardiovascular toxicities linked to anticancer drugs include increases of QT duration, arrhythmias, and myocardial ischemia (antimetabolite compounds) [6], hypertension and thromboembolic complications (antiangiogenic agents) [7], and myocardial dysfunction [1]. The latter, variable in severity, can be reversible or not and can occur during treatment or later on. For example, the clinical use of anthracyclines such as doxorubicin is hampered by the development of cardiomyopathy and congestive heart failure, which are typically dose-dependent and cumulative. While acute cardiotoxicity occurs, the most troublesome form manifests late after treatment and is characterized by structural changes of the human heart [8], leading to decreases in the left ventricle wall thickness and myocardium mass, as well as reduced ventricular compliance [9]. Unlike acute toxicity, the delayed manifestation of anthracycline use often presents as symptomatic heart failure and is considered largely irreversible [1, 5, 10]. 

Although past studies of cardiotoxicity have focused on anthracyclines, more recently interest has turned to anticancer drugs that target many proteins kinases, such as tyrosine kinases [2]. Targeted therapeutics, particularly those that inhibit the activity of protein kinases that are mutated and/or overexpressed in cancer, have revolutionized the treatment of some cancers and improved survival in many others [11, 12]. Unexpected cardiotoxicity induced by targeted drugs has been related to the existence of numerous parallels between signaling pathways that drive tumorigenesis and those that regulate survival of cardiomyocytes [12–15]. For example, on-target heart toxicity of trastuzumab, a monoclonal antibody against the ErbB2 receptor [16] revealed that human epidermal growth factor receptor 2 signaling also interfered with survival pathway in cardiomyocyte, a terminally differentiated cell [16, 17]. At this point, it can be hypothesized that mitochondrial dysfunction and ATP depletion are the main contributors to targeted therapy-induced cardiac toxicity [15].

An attractive model to explain the mechanism of this cardiotoxicity could be myocyte loss through cell death pathways [18, 19]. Given the limited regenerative capacity of the heart, cumulative toxicity may be explained by the progressive increase of cardiac cell loss. Cardiac cell stress (specifically oxidative stress induced by anthracyclines and many kinase inhibitors) activates apoptosis and necrosis via a mitochondrial pathway [1, 10]. As mitochondria are a central component of intrinsic apoptotic and necrotic pathways, mitochondrial “effects” of anticancer drugs are to be an expected outcome of adverse interactions between the drug and cells [20].

## 2. Overview of Anthracycline Cardiotoxicity

### 2.1. Clinical Picture of Cardiac Toxicity

Anthracycline-induced cardiotoxicity has been categorized into acute, early-onset chronic progressive and late-onset chronic progressive forms [21]. Acute cardiotoxicity occurs during or shortly after drug infusion and includes nonspecific EKG changes and arrhythmias, which may be accompanied in some patients by heart failure and pericarditis-myocarditis syndrome [9]. These complications are typically reversible, not dose-dependent and do not preclude further anthracycline use. Single cases of acute cardiac failure and sudden death were also reported [9, 22]. The subacute cardiac toxicity occurs within a few weeks, clinically resembles myocarditis (with edema and thickening of the left ventricle LV walls), and is associated with 60% mortality [22]. Acute cardiotoxicity occur in 1% of patients, whereas the subacute form occurs in 1.4–2% of patients [9]. Clinically the most significant effect of anthracyclines is chronic cardiac toxicity that may lead to LV dysfunction and congestive heart failure [23, 24]. Late-onset chronic progressive cardiotoxicity usually appears at least one year after completion of therapy and manifests clinically in 1.6–5% of patients [24]. Late-onset chronic progressive may not become evident until 10 to 20 years after the first dose of cancer treatment. The prognosis in anthracycline-related heart failure is poor, with 50% 2-year mortality in untreated established LV dysfunction [24, 25]. The most important risk factor for late cardiac toxicity is the cumulative anthracycline dose [25].

It is believed that each anthracycline dose causes structural changes to cardiomyocytes, which ultimately lead to cardiomyocyte death. These defects are balanced by compensatory mechanisms until a certain threshold, above which ventricular remodeling common to multiple forms of cardiac injury is triggered. The estimated risk of chronic heart failure for doxorubicin dose greater than 400 mg/m^2^ ranges from 3% to 5%, for 550 mg/m^2^ from 7% to 26%, and for 700 mg/m^2^ from 18% to 48% [1, 10]. In clinical practice, however, it seems wise to estimate the risk as being in the upper limit of the given ranges and adapt maximum cumulative doses accordingly [26]. Despite a considerable variability in individual dose-response relationship for cardiac toxicity, the maximum lifetime cumulative dose for doxorubicin is 400 to 550 mg/m^2^ [1, 10]. The long-term follow-up data of patients treated with adjuvant anthracycline-based regimens demonstrated increased incidence of symptomatic and asymptomatic left ventricular dysfunction and decrease in the mean LVEF value, suggesting that the incidence and severity of postanthracycline heart damage increases with time [26]. Overall, these data stress the importance of cardiac follow-up well beyond the treatment period.

### 2.2. Proposed Pathophysiology

**Figure 1:**
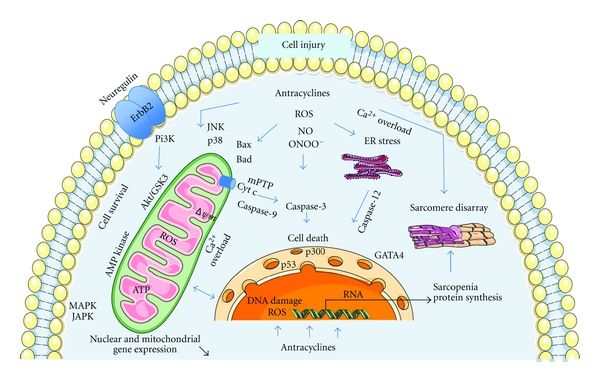
Potential signaling pathways involved in anthracycline-induced cardiomyocyte injury. Anthracycline-induced cell death is balanced by intracellular survival signaling which is linked to neuregulin/ErbB2 and Akt activation. The suggested principal mechanism of anthracycline damage is via generation of reactive oxygen species ROS by iron-anthracycline complexes, leading to lipid peroxidation and membrane damage. Oxidative stress (ROS, nitric oxide NO, and peroxynitrite ONOO–) causes activation of kinase pathways (mitogen-activated protein kinase MAPK, stress-activated protein kinase SAPK, c-Jun N-terminal kinases JNK) modulating response to anthracyclines and linking to apoptotic pathway. In mitochondria, ROS and calcium overload lead to the release of cytochrome c (cyt c) from mitochondria into cytoplasm, via mitochondrial permeability transition pore opening (mPTP), which results in membrane potential dissipation (delta psi m), activation of caspases and apoptosis. Other putative mechanisms include damage to nuclear DNA, disruption of sarcomeric protein, suppression of transcription factors (GATA-4, p300, p53) that regulate cell survival and sarcomeric protein synthesis, and disturbance of energy metabolism.

Despite a remarkably extensive literature on many aspects of cardiotoxicity, a single unifying theory for the deleterious effects of anthracyclines on the heart is still lacking. Anthracycline-induced myocardial damage has long been regarded as occurring primarily through the generation of reactive oxygen species and free radicals [27]. This oxidative stress model is supported by many studies showing ROS formation, especially in the setting of increased intracellular iron levels, in response to anthracycline treatment [27, 28]. Recent studies, however, have suggested that the ROS model is inadequate to account for all features of anthracycline cardiotoxicity [27]. Indeed, there is strong evidence that anthracycline cardiotoxicity stems from (at least partially) ROS-independent mechanisms, such as cardiomyocyte apoptosis or necrosis, disruption of normal sarcomere structure and altered energetics impairing the cardiac cell ability to generate adequate contraction [18, 29–31]. Recent studies have highlighted the effects of anthracyclines on compensatory prosurvival mechanisms, such as neuregulin/heregulin-Erb/HER2 and cell salvage kinase pathways, which may modulate the development of heart failure [32, 33].  Figure 1 provides a summary of the different signaling pathways involved in anthracycline-induced cardiotoxicity.

### 2.3. Oxidative Stress Pathway and Iron Hypothesis

The chemical structure of anthracyclines is complex: these drugs are composed of an aglycone and a sugar. The aglycone consists of a tetracyclic ring with adjacent quinone-hydroquinone moieties. Quinone moieties have toxicological importance because of their involvement in both reductive and oxidative biotransformation leading to highly reactive species involved in cardiotoxicity [27, 34].

One-electron reduction of the tetracyclic ring of anthracyclines leads to the formation of a semiquinone free radical. This radical is relatively stable in an anoxic environment, but under normoxic conditions, its unpaired electron is donated to oxygen, forming superoxide radicals. Suitable flavoproteins such as complex I catalyze the formation of reduced semiquinone radicals by accepting electrons from NADH or NADPH and donating them to anthracyclines. This sequence of reactions, known as “redox cycling,” can be highly damaging, because a relatively small amount of drug is sufficient for the formation of numerous superoxide radicals. The redox cycling of anthracyclines has been described in cytoplasm, mitochondria, and sarcoplasmic reticulum [27]. The first targets of anthracycline-mediated free-radical damage are various cellular membranes, which are rich in lipids prone to peroxidation. This radical damage results in production of many stable and highly toxic aldehydes, which further attack macromolecular targets. Although formation of ROS is induced by the quinone moiety of anthracyclines, oxidative stress can also occur via induction of nitric oxide synthase, leading to superoxide anion, nitric oxide and peroxynitrite formation [18, 27].

Promotion of myocardial oxidative stress remains by far the most frequently proposed mechanisms of anthracycline-induced cardiotoxicity [27]. Production of ROS that follows nuclear binding of the drug results in injuries to DNA as well as to cell membranes and mitochondria. ROS production is involved in a vast variety of cardiotoxicity inducing mechanisms, including impaired expression of cardiac proteins, disruption of cellular and mitochondrial calcium homeostasis, induction of mitochondrial DNA lesions, disruption of mitochondrial bioenergetics and ATP transfer systems, and degradation of myofilament and cytoskeleton proteins [18, 29–31]. Involvement of oxidative stress in the pathogenesis of anthracycline cardiotoxicity has been supported by several approaches, that is, isolated cardiac cells displaying perimitochondrial ROS production in response to anthracycline exposition, cultured cell and animal models showing that antioxidant prevented anthracycline-induced cardiotoxicity, and resistance of transgenic mice overexpressing the mitochondrial manganese-superoxide dismutase to anthracycline-induced cardiotoxicity [35].

Anthracyclines may promote the formation of ROS through redox cycling of their aglycones as well as their anthracycline-iron complexes. Indeed, unless adequately sequestered within the cells, iron can dramatically promote ROS production by the Fenton and Haber-Weiss reactions and the formation of reactive anthracycline-iron complexes [27, 34]. Cellular iron (Fe) level is tightly regulated by the transferring receptor and storage regulating ferritin, both of which are, themselves regulated at the posttranscriptional level by interactions of Fe-regulatory-protein (IRP-1) with specific motifs iron-responsive-elements (IREs) in target genes. Doxorubicin and its metabolites can disrupt the Fe-S cluster of cytoplasmic aconitase and inhibit IRP-1. In doxorubicin-treated cardiomyocytes, increased IRP-1 inhibition leads to intracellular Fe accumulation causing increased oxidative stress [36].

Consistent results suggesting the involvement of oxidative and nitrosative oxidant stresses in anthracycline-induced cardiotoxicity provide a rationale for cardioprotection with antioxidants in humans [27, 37]. Unfortunately, use of different antioxidant agents have failed to provide protection in both preclinical experiments and clinical studies [3]. First generation antioxidant molecules such as N-acetylcystein, vitamins D, E have been investigated on the basis of some protective effects observed in animal models [27]. None of these antioxidant approaches has yet shown consistent efficiency. The only compound found to reduce long-term cardiac dysfunction is dexrazoxane, although whether its efficiency is related to its antioxidant properties or other mechanisms remains under debate [3]. For example, underlying mechanisms of dexrazoxane include prevention of iron accumulation, which is implicated in increased ROS production and anthracycline-induced cardiotoxicity.

### 2.4. Mitochondrial Apoptosis and Necrosis Pathway

Cardiac side effects of anthracyclines involve two main mechanisms, which interact with each other, oxidative stress and apoptosis [18, 27]. Most of the cellular events induced by ROS generation contribute to cardiomyocyte death, which has been shown to be a primary mechanism for anthracycline-induced cardiomyopathy. Indeed, cardiac myocyte loss following activation of both apoptotic and necrotic pathways provide an attractive explanation for anthracycline-induced cardiotoxicity [18, 20, 38]. Studies in animals have demonstrated that apoptotic cell death occurs after in vivo exposure to anthracyclines [38]. Experimental cell cultures have also shown that anthracyclines induce both apoptotic and necrotic cell death [38]. Evidence of mitochondrial injuries and hallmarks of apoptosis have been found in endomyocardial biopsies of patients treated with anthracyclines [8, 39]. Overall, cardiac myocyte death following anthracycline administration typically presents with biochemical features of apoptosis and the morphological aspect of cell necrosis [18, 20, 38].

Anthracycline-induced apoptosis in the heart appears to involve a mitochondrial pathway, which requires Bax, cytochrome c and caspase-3 [20]. Typically, anthracycline treatment increases mitochondrial oxidative stress and disrupts intracellular calcium levels [40, 41]. Increased intracellular calcium, favored by calcium flux aberrations, eventually raises mitochondrial calcium levels. Above a certain threshold, this calcium overload triggers permeability transition of the mitochondria, resulting in the dissipation of transmembrane potential, as well as mitochondrial swelling and increased permeability of its outer membrane to apoptotic factors such as cytochrome c [20]. In the cytosol, cytochrome c forms a complex with the adaptor protein apoptosis protease activator protein-1 (Apaf-1), dATP, and caspase-9, so-called apoptosome, which in turn activates caspase-9. The intrinsic pathway converges then to the downstream executioner caspases [20].

The current hypothesis is that necrotic and some forms of apoptotic cell death involve prolonged opening of a large conductance pore in the mitochondria, known as the mitochondrial permeability transition pore mPTP [36]. In its fully open state, the mPTP has been reported to allow unrestricted movement of solutes of <1.5 kDa. The activation of the mPTP in isolated mitochondria has been shown to lead to mitochondrial swelling, which is commonly used as an assay for mPTP opening. In spite of the great recent interest concerning mPTP and its apparent importance in cell death, its molecular identity is unknown. It has been proposed that the mPTP is formed through conformational change in the association of the adenine nucleotide translocator (ANT) with the voltage dependent anion channel (VDAC) contact sites between the inner and outer mitochondrial membranes [42, 43]. Cyclophilin D is thought to regulate the opening of the pore via its interactions with ANT. These interactions are inhibited by cyclosporine A supporting the idea that cyclophilin D play a role in pore opening [42, 43]. Recent studies have shown that genetic ablation of either ANT or VDAC isoforms did not result in the absence of mPTP, suggesting that neither of these proteins is an obligatory component [43]. In contrat, ablation of cyclophilin D reduces ischemia-reperfusion-induced cell death, suggesting a role for cyclophilin in the mPTP [44, 45]. Growing evidence suggests that the phosphate carrier is a critical component of the mPTP and that interaction between ANT and the phosphate carrier can modulate mPTP opening [45].

For many years, it has been put forward that the mPTP contributed mainly to apoptotic cell death as a protagonist of mitochondrial permeabilization. Recent data suggests, however, that an increase in mitochondrial membrane permeabilization is one of the key events in both apoptotic and necrotic cell death [43, 44]. This information is important since necrosis occurs in many forms of adult human heart injuries, including the cardiotoxic effects of anticancer drugs. Indeed, in the mid-2000s, new experimental studies suggested that mPTP did not initiate apoptosis and that this complex instead played a central role in necrosis, especially in the heart. In this line of reasoning, it has been shown that cyclosporine A (CsA), a known inhibitor of the mPTP, can reduce the occurrence of cardiac and brain cell necrosis during ischemia reperfusion injury. Studies of cyclophilin-D-deficient mice have also provided consistent evidence that the mPTP plays a crucial role in cell necrosis [46, 47]. In these mice, mPTP was still functional but cyclophilin-D ablation increased the amount of calcium required for mPTP opening and abolished the sensitivity to CsA [46, 48]. Cyclophilin-D-deficient mice had increased resistance against necrotic stimuli such as calcium overload, whereas these animals still died in response to treatments with classical apoptotic inducers such as staurosporine or etoposide. Overall, this data indicates that mPTP opening is chiefly involved in cardiac cell necrosis rather than in triggering cytochrome c release during early apoptosis.

### 2.5. Alternative Types of Cell Death: Oncosis and Autophagy

Features of cell oncosis, which is typically associated in cardiomyocytes with mitochondrial and cytoplasmic swelling, coagulated sarcomere and early rupture of the plasma membrane [49] have been described in anthracycline-induced cardiac cell damage [50]. As mentioned above, recent studies have shown that this form of cell death can be well controlled and programmed through mPTP-dependent mechanisms. The rationale is that increased ROS leads to mitochondrial calcium overloading, promotes mPTP opening, causes mitochondrial swelling and ATP depletion, and hence triggers necrotic cell death [20]. Autophagy has evolved as a conserving process that uses bulk degradation and recycling of cytoplasmic components, such as long-lived proteins and organelles. In the heart, autophagy is important for the turnover of organelles at low basal levels under normal conditions and it is upregulated in response to stresses such as ischemia/reperfusion and in cardiovascular diseases such as heart failure. Recent evidence suggests that autophagic cell death may play a significant role in the myocardial dysfunction induced by doxorubicin [51]. Overall, it could be stated that in anthracyclin cardiotoxicity, mitochondria is the crossroad for apoptosis, necrosis and autophagy processes, which may converge in dying cells in response to different pathways including ROS production, calcium overload, and DNA lesions.

## 3. Mitochondria-Related Survival/Death Pathways

If apoptotic and necrotic cell death are central to the feature of anthracycline-induced cardiotoxicity, then the underlying mechanisms in play are worth exploring, as they may lead to cytoprotective strategies. Likewise, better understanding of activation of cell survival pathways in response to anthracycline exposition may also provide valuable knowledge that would help in the development of new cytoprotective strategies.

### 3.1. Mitochondrial Permeability Transition

Bioenergetic failure, enzyme inhibitions, lipid peroxidations, induction of membrane disorders as well as the initiation of oxidative stress are being attributed to the accumulation of anthracyclines at or inside mitochondria. From heart tissue perfused with anthracyclines two distinct cellular sites of drug accumulation were the nuclei and mitochondria, which become labeled with the drug [52, 53]. Hence, it has been commonly proposed that deleterious signals related to anthracycline exposure converge to the mitochondria to favor mPTP-mediated cell death [54]. In addition, if this hypothesis is correct, an understanding cardioprotective mechanisms is intimately linked to an understanding of the mechanisms by which mitochondria regulate cell death.

Disruption of mitochondrial calcium homeostasis following chronic doxorubicin administration can be demonstrated by using cardiac mitochondria isolated from doxorubicin-treated animals [40, 41]. For example, activation of the selective cyclosporine- (CsA-)sensitive calcium channel of cardiac mitochondria by doxorubicin occurs both in vitro [55] and in cardiac mitochondria isolated in rats having undergone chronic in vivo treatment with doxorubicin [56]. In the latter protocol of exposition (2 mg/kg/week doxorubicin treatment for 13 weeks), isolated mitochondria have a lower respiratory control ratio and exhibit an enhanced CsA-sensitive release of mitochondrial calcium. Associated with this was a calcium-induced loss of membrane potential, which may be inhibited by either cyclosporine A or ruthenium red. Further experiments have demonstrated that doxorubicin treatment in vivo causes a dose-dependent and irreversible interference of mitochondrial calcium transport and calcium-dependent regulation of membrane potential indicative of an induction of the mPTP and of an increased sensitivity to calcium-induced loss of cell viability [57, 58]. Implication of the mPTP in the cardiotoxicity of doxorubicin has been explored in cyclophilin-D-deficient mice, cyclophilin-D being a mitochondrial matrix peptidyl-prolyl isomerase known to modulated mitochondrial transition pore opening. The result that cyclophilin-D deficiency in mice inhibited doxorubicin-induced cardiomyocyte necrosis and heart failure suggests that mPTP is involved in doxorubicin-induced cardiotoxicity [48]. This contention is also supported by animal studies showing prevention of doxorubicin cardiotoxicity by in vivo CsA or FK506 treatment [59, 60]. In human atrial trabeculae, our group also demonstrated that cyclosporine A prevented doxorubicin-induced mitochondrial dysfunction and impaired contractile performance induced by doxorubicin [61]. These findings reinforce the rationale that mPTP is involved in the development of doxorubicin cardiotoxicity in the human myocardium.

### 3.2. Survival Protein Kinase Signaling

Accumulating evidence indicates that several protein kinases (i.e., Akt, PKCs, EKR, GSK-3b, hexokinase) receive extra mitochondrial signals and modify mitochondrial proteins that determine cell death or survival, such as the mPTP [62]. Activities of some of these kinases are mutually regulated, and phosphorylation of GSK-3*β* and hexokinase in mitochondria appears to directly modify the mPTP, elevating its opening threshold [62]. Doxorubicin may induce inhibition of Akt phosphorylation, which increases active glycogen synthase kinase-3b (GSK-3b) [63]. GSK-3b is a protein kinase linked to the regulation of a variety of cellular functions within the myocardium, including glycogen metabolism, gene expression and cellular survival [62]. GSK-3b phosphorylation, and, therefore inhibition, could confer cardioprotection through its potential mitochondrial effects on the mPTP. Strategies that prevent GSK-3b activation via upstream kinase activation have been shown to be protective against doxorubicin treatment. For example, pretreatment with various therapeutic molecules (erythropoietin, thrombopoietin, CO/HO1) can protect the myocardium against doxorubicin-induced impaired heart function and cardiomyocyte apoptosis by activating Pi3k-Akt cell survival pathways [63–65]. In contrast, upregulation of Ser/Thr phosphatase PP1 by doxorubicin may be involved in the Akt dephosphorylation, resulting in executioner caspase activation and cell death [66].

Cellular stress and specifically oxidative stress has been shown to activate mitogen/stress activated protein kinases (MAPKs and SAPKs) that appear to be important in determining cell fate. MAPKs and SAPKs pathways modulate the response of the heart to anthracycline exposure [67] and have also been proposed as cellular mediators linking anthracyclines to the apoptotic cell death pathway [18, 30]. Under treatment with anthracyclines that significantly induces myocyte apoptosis in the primary cultures of neonatal mouse cardiomyocytes, p38 MAPK is dramatically activated. That p38 MAPK may be involved at least in part in the anthracycline-induced myocyte apoptosis is demonstrated by two important observations. First, the time-course analysis revealed that p38 MAPK activation typically precedes the onset of apoptosis. Second, application of inhibitors of p38 MAPK significantly inhibits anthracycline-induced myocyte apoptosis [68]. Although most studies have focused on the ERK member of the MAPKs, other members of the MAPKs/SAPKs family have been associated with cardioprotection through the modulation of the mPTP. For example, JNK and p38 are activated by doxorubicin and linked mPTP to cardiac myocyte apoptosis [69]. Strategies reducing activation of the MPAKs/SAPKs pathways are typically protective against doxorubicin cardiotoxicity.

### 3.3. Genomic Analyses and Cellular Energetic Deficits

Doxorubicin typically causes selective downregulation of many nuclear genes that encode for proteins with mitochondrial function [70, 71]. The depressive effect on the expression of genes that comprise the mitochondrial proteome is persistent and can be observed weeks after prolonged administration of doxorubicin [70]. Previous studies suggest that a prominent feature of doxorubicin-induced cardiotoxicity is a profound alteration in the abundance of transcripts related to energy metabolism and mitochondrial performance. Consistent evidence suggests that cellular energy deficits related to decrease in fatty acid and glucose oxidation could play a critical role in the development of the cardiomyopathy induced by anthracyclines [29]. For example, oxidation of the long-chain fatty acid palmitate is inhibited by doxorubicin within minutes in isolated cardiomyocyte preparation, as well as in chronic situation in which cardiomyocytes are isolated from doxorubicin-treated rats. In these studies, impairment of carnitine palmitoyl transferase I and depletion of its substrate l-carnitine by doxorubicin was demonstrated [72]. Reduction in fatty acid oxidation is not accompanied by upregulation of glucose utilization as a compensatory response [73]. Rather, doxorubicin-induced cardiomyopathy is associated with a decreased utilization of both fatty acids and glucose substrates, which has been related to the effects of doxorubicin on cellular glucose supply [74] and the impairment of phosphofructokinase, the rate-limiting enzyme of glycolysis [75].

Furthermore, the selective effects of doxorubicin on suppression of mitochondria gene expression is accompanied by a coordinate and adaptive response of energy-sensing molecules [76], such as AMP-activated protein kinase (AMPK), hypoxia-inducible factor 1 (HIF1), nuclear respiratory factors (Nrf) and proliferator-activated receptor gamma coactivator1 (PGC1) [77]. Proteomic analyses revealed consistent changes in proteins involved in mitochondria energy production, energy channeling and mitochondrial antioxidant protection [78, 79]. Overall, this information is in line with doxorubicin-induced mitochondrial defects at different stages of cardiac energy metabolism, including reduction of oxidative capacity, changes in the profile of energy substrate utilization, disruption of energy transfer systems such as mitochondrial CK, and AMPK-dependent energy signaling pathways.

### 3.4. Anthracyclines Induce Sarcomere Functional and Structural Changes

Functional and structural changes to cardiomyocyte sarcomeres have been observed in both experimental experiments and in endomyocardial biopsies of patients treated with anthracyclines. Loss of myofibrils, disarray of myofibrils, swelling of mitochondria and dilation of the sarcoplasmic reticulum were observed [80, 81]. Breakdown of sarcomeres typically involved early-onset degradation of the giant myofilament protein, titin. As titin maintains sarcomere integrity, its accelerated degradation via calpain proteolytic activity in response to doxorubicin can rapidly lead to sarcomere disorganization and progressive cardiomyocyte contractile dysfunction [82].

Several lines of evidence indicate that an abnormal calcium handling of myocardial cells may explain, at least in part, the cardiac dysfunction seen in doxorubicin-induced cardiomyopathy. For example, doxorubicin has been shown to inhibit the gene transcription of the sarcoplasmic reticulum Ca2+-ATPase [83] and to activate cardiac calcium release channels (ryanodine receptors) [84]. A decrease of sarcoplasmic reticulum calcium load and hence calcium-induced calcium release has been observed with doxorubicin in isolated guinea pig ventricular myocytes [85]. The mechanism by which doxorubicin affects calcium homeostasis of cardiac myocytes has not been fully defined but may involve an iron-catalyzed direct effect of doxorubicin, doxorubicin-induced formation of reactive oxygen intermediates [83], and conversion of doxorubicin to the toxic alcohol metabolites [86].

### 3.5. Transcriptional Factors

Several lines of evidence suggest that progressive anthracycline-induced cardiac injury results from effects on myocyte differentiation programs thereby impeding myocyte survival and the cardiac adaptative response. Genes with anthracycline inhibited expression include genes encoding, transcriptional factors [71, 73]. For example, anthracyclines can disrupt expression and activity of the transcription factor GATA-4 [87]. Transcriptional factor GATA-4 is a member of a zinc finger transcriptional factor family that is critical for regulating differentiation, sarcomere synthesis and survival signaling. GATA-4 is expressed in the heart and regulates several specific cardiac genes, including antiapoptotic genes, making it a key regulator of heart development. This important survival factor is rapidly depleted in response to doxorubicin treatment [64, 88]. Anthracyclines downregulate GATA-4 expression in cardiac myocytes and upregulation of GATA-4 can suppress doxorubicin-induced myocyte apoptosis and drug-induced cardiotoxicity [88, 89]. These prosurvival effects have been linked to the effects of GATA-4 on the upstream activator of the antiapoptotic gene Bcl-Xl. Since the overexpression of GATA-4 can attenuate the incidence of apoptosis induced by anthracyclines, GATA-4 may serve as an antiapoptotic factor in the heart. Moreover, GATA-4 also regulates expression of several cardiac specific genes that are involved in sarcomere synthesis, such as cardiac troponin C and I and myosin light chain-3 [90]. Hence, one potential mechanism by which anthracycline may induced myocardial dysfunction is via suppression of sarcomere protein expression and sarcopenia in response to GATA-4 reduction [91].

In addition to GATA-4, the cardiac ankyrin repeat protein transcriptional regulator CARP and the transcriptional coactivating factor p300 have been implicated in the cardiotoxicity of anthracyclines. CARP is rapidly degraded in myocytes after anthracycline exposure [91, 92]. Suppression of CARP expression using short-interference RNA is sufficient to induce myofibrillar disarray and cell dysfunction [93]. Similar to CARP, p300 is degraded after doxorubicin exposure through p38 kinases alpha and beta and is associated with apoptosis in neonatal cardiomyocytes [93, 94]. In these experiments, restoration of p300 inhibited doxorubicin-induced cell death.

Doxorubicin treatment has been associated with increased expression and activation of p53 tumor suppressor protein, which activates the intrinsic mitochondrial apoptotic pathway [95]. Consistently, p53-knockout mice and adult mouse hearts expressing cardiac myocyte-restricted dominant-interfering p53 are partially protected against doxorubicin-induced cell death and myocardial dysfunction [96]. In addition, activation of p53 may also mediate anthracycline-induced cardiotoxicity through other pathways independent of cardiomyocyte apoptosis. For example, p53-mediated inhibition of mammalian target of rapamycin signaling (mTOR) may contribute to the cardiac mass reduction and myocardial dysfunction observed in doxorubicin-treated mice [96]. Hence, acute doxorubicin-induced toxicity could result from p53-dependent modulation of mTOR activity. It may be thus of considerable interest to determine whether upstream effectors that activate mTOR pathway would be cardioprotective against doxorubicin-induced cardiac toxicity.

### 3.6. Neuregulin/ErbB2 Cardioprotective Program

Unexpected cardiac side effect of ErbB2 antagonists, such as trastuzumab, has sparked great scientific efforts to elucidate the role of Neuregulin/ErbB2 pathway in cardiomyocyte functional and structural integrity [16, 17, 97]. Before this observation, ErbB2 signaling was only recognized as being indispensable to normal fetal cardiac development. Subsequent studies have demonstrated that stimulation of the ErbB2 signaling by ErbB-receptor ligands improves cardiomyocyte function and survival in the heart [97].

The first evidence regarding the protective effects of the ErbB2 signaling in the adult heart came from clinical trials in breast cancer patients using trastuzumab, a monoclonal antibody that blocks the ErbB2 receptor [1, 2]. The incidence of clinical heart failure increased five-fold in patients treated concurrently with chemotherapy drug doxorubicin and trastuzumab compared to those treated with doxorubicin alone [1]. Mostly based on the analogy between ErbB2 knockout-induced cardiomyopathy and trastuzumab-induced heart failure, many studies have concluded that trastuzumab causes heart failure by blocking the physiological actions of ErbB2 in the heart [32, 33]. The synergistic increase of heart failure incidence has been related to the fact that ErbB2 expression is upregulated following doxorubicin administration, while trastuzumab inhibits the ErbB2 downstream pathways, which is essential for cell repair, survival, and function. Thus, if trastuzumab inhibits the ErbB2 cardioprotective pathways during a vulnerable period after anthracycline injury, the anthracycline damage could be augmented, resulting in increased cell death [2].

Overall, these results suggest that ErbB2 inhibition result in mitochondrial apoptotic signaling in cardiomyocytes leading to increased cell loss in the heart. Therefore, up-regulation of the cardiac neuregulin/ErbB2 pathway may be one strategy to limit myocardial anthracycline injury. Experimental work on both animals and humans has demonstrated that exercise is a potent activator of neuregulin release with subsequent activation of ErbB2 activation [98]. The fact that exercise protects against calcium-induced cardiac mitochondrial permeability transition and reduces cell death following doxorubicin administration could be related to the neuregulin/ErbB2 survival pathway activation [99].

### 3.7. Lessons from Targeted Chemotherapy

Several novel “targeted” agents have been associated with a small but worrisome risk of heart dysfunction [2, 12–14]. These agents include the tyrosine kinase inhibitors sunitinib, lapatinib, and imatinib, which are members of a growing class of targeted chemotherapy agents. Clues as to the nature of the cardiotoxicity due to these agents are beginning to emerge that point to the mitochondria. To date, the only approved kinase inhibitor that is clearly associated with clinical cardiotoxicity is sunitinib, whereas the extent of imatinib-induced cardiotoxicity is still under scrutiny [12].

On-target cardiac toxicity is inherent to kinase inhibition and quickly becomes apparent since many of the pathways that regulate cancer cell survival also regulate essential processes in cardiomyocytes, including contractile function and survival [14, 17]. The ATP binding pocket represents the key region of the kinase targeted by most inhibitors. Conservation of that ATP binding pocket among kinases means that inhibitors can also inhibit unintended kinases, and if any of these kinases serve important functions in the heart, off-target cardiotoxicity can occur. An additional issue is that targeting of kinases will achieve entire pathway targeting. For example, the inhibition of multiple components of the Pi3kinase/Akt pathway is a viable strategy for cancers, but this pathway also maintains cardiomyocyte homeostasis and protects cardiomyocytes from death [100]. Eventually, kinase inhibitors could mediate toxicity through the inhibition of non-kinase targets, such an enzyme requiring ATP to perform its function [12, 13].

To illustrate the complexities inherent in identifying mechanisms of kinase inhibitor-induced cardiac toxicity, sunitinib-induced cardiac injury will be discussed. Cardiac dysfunction was first related to systemic hypertension secondary to VEGFR2 and PDGFRbeta inhibition by sunitinib [101]. As several patients developed cardiotoxicity in absence of hypertension, additional mechanisms were explored. In this study, endomyocardial biopsy samples from two patients who presented with profound sunitinib-induced heart failure were obtained. Abnormal histopathological changes included marked mitochondrial swelling, which could indicate mPTP and energetic failure [101, 102]. In cultured cardiomyocytes, the same mitochondrial abnormalities were observed and were associated with apoptotic cell death [101, 102]. Studies in cardiomyocytes have confirmed that energy compromise was involved in sunitinib-induced cardiac toxicity but surprisingly was not associated with activation of AMP kinase, the master fuel control of the cell [102]. Lack of response to this energy loss was the result of direct inhibition of AMP kinase by sunitinib, so-called off-target toxicity. AMP kinase inhibition has also been reported in isolated heart exposed to doxorubicin [73], whereas recent studies demonstrated that AMPK gene expression and enzyme activities were acutely increased [71]. These results are important since AMPK is implicated in many survival pathways, including Pi3k/Akt/mTOR axis [103].

Likewise, imatinib causes a modest but consistent decline in left ventricular function, which was associated with loss of myocardial mass and increased cell death [104]. Studies in cardiomyocytes showed that imatinib leads to significant mitochondrial dysfunction with mitochondria swelling, mitochondrial membrane potential collapse followed by cytochrome c release and energetic failure [15, 104]. This process was associated with a cell death that has the biochemical features of apoptosis and the morphological aspect of cell necrosis. Mitochondria isolated from hearts of mice treated with imatinib showed enhanced calcium-induced swelling and mitochondrial permeability transition mPTP. As is the case with trastuzumab, mitochondrial dysfunction plays a central role in the cardiotoxic response to imatinib, but the mechanism seems to be the induction of endoplasmic reticulum ER stress by the drug [15, 104]. ER stress in response to imatinib exposition has been related to the downstream activation of the c-Jun N-terminal kinase (JNK) family of stress MAPKs [105]. Similarly, ER stress-mediated apoptotic pathway has also been reported to mediate cardiac cell death induced by doxorubicin [20]. In this case, caspase 12, which resides in the ER, is an essential caspase to initiate ER-mediated apoptosis that is activated in doxorubicin-treated hearts [106].

The Pi3k/Akt/mTOR pathway more than any other epitomizes the similarity between cancer cell signaling and survival signaling in cardiac cells [54]. The cautionary note is that Pi3k/Akt/mTOR pathway is also critical for cardiomyocyte integrity and survival. Hence, inhibition of multiple components of the pathway would jeopardize cardiomyocyte integrity and survival [100]. Conversely, activation of this signaling cascade, together with other Akt-activated molecules (such as GSK-3*β*, mTOR and p70S6 kinase), elicits prosurvival and cardiovascular protective effects, which are mediated by inhibition of opening of the mPTP [62].

## 4. Conclusion

Cardiac dysfunction is the most severe side effects of anthracycline treatment. The major mechanism of anthracycline damage involves the generation of reactive oxygen species ROS by iron-anthracycline complexes, leading to lipid peroxidation and membrane damage. In mitochondria, ROS and calcium overload lead to mitochondrial permeability transition pore opening (mPTP), which is associated with the release of cytochrome c (cyt c) from mitochondria into cytoplasm and cell death. Cardiac myocyte loss following activation of both apoptotic and necrotic pathways provide an attractive explanation for anthracycline-induced cardiotoxicity. There is evidence that the stressed cardiac myocyte survival may rely on both growth and survival pathways that are altered in the anthracycline-exposed myocardium. Activation of ErbB2 and Pi3k/Akt/mTOR pathways represents a major adaptative mechanism for cardioprotection, which are altered by anthracyclines. The delicate balance between pro- and antiapoptosis signaling that relies on kinase-regulated pathways creates a cause for concern when one attempts to use anticancer molecules, that is, targeted therapies that can impair the coordinated function of this kinase network.

## Abbreviations

(HER2 or EbrB2): Human epidermal growth factor receptor 2
Pi3k: Phosphatidylinositol-3-Kinase
Akt: Serine/threonine protein kinase
GSK3: Glycogen Synthase Kinase 3;
BCL: B Cell Lymphoma
Bax: (BCL)-associated X
ER stress: Endoplasmic reticulum
GATA: Family name is derived from their ability to bind to the consensus DNA sequence (A/T) GATA (A/G)
p53: Tumor suppressor p53
p300: Transcription factor p300
ΔΨ: Mitochondrial membrane potential.
